# Fiscal austerity and municipal health spending: an interrupted time series study

**DOI:** 10.11606/s1518-8787.2024058005772

**Published:** 2024-09-16

**Authors:** Leo Ramos Maia, Mônica Rodrigues Campos, Débora Castanheira

**Affiliations:** I Fundação Oswaldo Cruz Escola Nacional de Saúde Pública Sergio Arouca Programa de Pós-Graduação em Saúde Pública Rio de Janeiro Brasil Fundação Oswaldo Cruz. Escola Nacional de Saúde Pública Sergio Arouca. Programa de Pós-Graduação em Saúde Pública. Rio de Janeiro, Brasil; II Fundação Oswaldo Cruz Escola Nacional de Saúde Pública Sergio Arouca Departamento de Ciências Sociais Rio de Janeiro Brasil Fundação Oswaldo Cruz. Escola Nacional de Saúde Pública Sergio Arouca Departamento de Ciências Sociais. Rio de Janeiro, Brasil; III Fundação Oswaldo Cruz Instituto Nacional de Infectologia Evandro Chagas Rio de Janeiro Brasil Fundação Oswaldo Cruz. Instituto Nacional de Infectologia Evandro Chagas. Rio de Janeiro, Brasil

**Keywords:** Financing Health Systems, Fiscal Policy, Public Spending on Health, Cities

## Abstract

**OBJECTIVE:**

To analyze the impact of the fiscal austerity policy (PAF) on health spending in Brazilian municipalities, considering population size and source of funds.

**METHODS:**

The interrupted time series method was used to analyze the effect of the PAF on total expenditure, resources transferred by the Federal Government, and own/state *per capita* resources allocated to health in the municipalities. The time series analyzed covered the period from 2010 to 2019, every six months. The first semester of 2015 was adopted as the start date of the intervention. The municipalities were grouped into small (up to 100,000 inhabitants), medium (101,000 to 400,000 inhabitants), and large (over 400,000 inhabitants). The data was obtained from the *Sistema de Informações sobre Orçamentos Públicos em Saúde* (Information System on Public Health Budget).

**RESULTS:**

The results for the national average of municipalities show that the PAF had a negative impact on the level of total expenditure and own/state resources allocated to health in the first half of 2015, without causing statically significant changes in the trends of any of the indicators analyzed in the period after 2015. Small municipalities saw a drop in total expenditure, while large municipalities saw a drop in own/state resources, and medium-sized municipalities saw a drop in both variables. There was no statistically significant drop in the volume of funds transferred by the Federal Government in the immediate aftermath of the implementation of the PAF in any of the municipal groups analyzed. In the medium-term, the PAF only had a negative impact on the large municipalities, which saw significant reductions in the trends of own/state resources and those transferred by the Union for health.

**CONCLUSION:**

In general, the impact of the PAF on health financing in municipalities was immediate and based on the decrease in own/state resources allocated to health. In large municipalities, however, the impact lasted from 2015 to 2019, mainly affecting health expenditure from federal funds.

## INTRODUCTION

In 2015, Brazil began implementing a fiscal austerity policy (PAF)^1^, defined as a reduction in public spending in subsequent years^2^. Its subsequent constitutionalization, through Constitutional Amendment No. 95 of 2016, has influenced the development of several studies focused on its effects on the health conditions of the population and on the provision of services and financing capacity of the Unified Health System (SUS)^3^.

In 2015, Brazil began implementing a fiscal austerity policy (PAF)^1^, defined as a reduction in public spending in subsequent years^2^. Its subsequent constitutionalization, through Constitutional Amendment No. 95 of 2016, has influenced the development of several studies focused on its effects on the health conditions of the population and on the provision of services and financing capacity of the Unified Health System (SUS)^3^.

In 2015, Brazil began implementing a fiscal austerity policy (PAF)^1^, defined as a reduction in public spending in subsequent years^2^. Its subsequent constitutionalization, through Constitutional Amendment No. 95 of 2016, has influenced the development of several studies focused on its effects on the health conditions of the population and on the provision of services and financing capacity of the Unified Health System (SUS)^3^.

Public spending *per capita* on health in Brazil, which had been on an upward trend since 2003, saw an unprecedented decrease in 2015^8^, the date that marked the beginning of the implementation of the PAF. Subsequently, public spending on health stagnated until 2019^9^, when, due to the COVID-19 pandemic, it was decided to break the spending ceiling and adopt an expansive fiscal policy^10^.

Cutting public spending on health means reducing the quality and quantity of health goods and services offered to the Brazilian population^6^, a reality that especially penalizes the poorest population, who depend on public services.

Studies evaluating the effects of the PAF on SUS financing focus mainly on total public and/or federal spending^7,9,11^. Regarding municipal spending on health, it is only known that, on the national average, it increased by 124% between 2004 and 2014, decreased by 9% in 2015, and gradually recovered between 2015 and 2019 (14%)^5^.

It is important to analyze the impact of the PAF on municipal health spending because the constitutional pact on decentralization placed a large part of the SUS’s responsibilities in the municipalities, the main one being Primary Health Care (PHC), which is a national management model^12^. In addition, the aforementioned growth in total public health expenditure in the period prior to the PAF was due to the increase in municipal participation in SUS funding^10^, a dynamic influenced by Constitutional Amendment No. 29 of 2000^13^.

It should also be borne in mind that the origin of resources and the volume of municipal expenditure on health are unequal according to municipal size^5^, because population groups are associated with the capacity to collect revenue, attract human resources and the infrastructure of the health system^14^, factors which influence the capacity and composition of expenditure^5^. Detailed analyses at this level of disaggregation are lacking in the literature.

With this in mind, the aim of this article is to analyze the impact of the PAF on health expenditure in Brazilian municipalities, taking into account population size and source of funds.

## METHODS

### Design and Fonts

This is a retrospective, quantitative, and analytical study that used the Interrupted Time Series (ITS) data regression method^15^. The *Sistema de Informações sobre Orçamentos Públicos em Saúde* (SIOPS – Information System on Public Health Budget)^16^ was used as a source, as well as population data from the demographic census (2010) and projections from the *Instituto Brasileiro de Geografia e Estatística* (IBGE – Brazilian Institute of Geography and Statistics).

Data from the SIOPS platform is not available in database format^16^, which is why the data used in this analysis was requested via the Access to Information Act. Data was found to be incomplete (an average of 10% over the period), especially between 2010 and 2013.

The municipalities were grouped into three population sizes: small (up to 100,000 inhabitants), medium (100,001 to 400,000 inhabitants), and large (400,001 or more inhabitants), considering residents in 2019. Brasilia was not included in the analysis because its sources of funding are different from those of the municipalities.

To characterize each group, indicators of socio-economic aspects and the health system were selected: 1) Life expectancy at birth; 2) Municipal Human Development Index (MHDI); 3) Degree of urbanization; 4) Number of doctors per 1,000 inhabitants; 5) Potential primary care coverage (COBAB)^17^; and 6) Complementary health coverage, which expresses the percentage of the population benefiting from private health plans.

The indicators come from the following sources: (1), (2), and (3) - estimates from the United Nations Development Program (UNDP) based on the 2010 demographic census; (4) - the System for the National Registry of Health Establishment (SCNES) and IBGE population projections; (5) - the Primary Care Information and Management System (e-Gestor AB); and (6) - the National Supplementary Health Agency (ANS).

### Outcome Indicators

Three indicators related to health financing from 2010 to 2019 were estimated every six months:

1. Total health expenditure: represents total settled health expenditure (excluding pensions), including that financed by other spheres of government, per inhabitant^18^.
2. Resources transferred by the Federal Government: represents the total amount of health revenues transferred by the Federal Government, per inhabitant.
3. Own and state resources: represents the total paid health expenditure (excluding pensions), minus the resources transferred by the Union, per inhabitant.

The national level of outcome indicators was estimated from the arithmetic mean of the three groups of municipalities.

All the values have been adjusted for the loss of inflation based on the Special Extended National Consumer Price Index (IPCA-E), supplied by the IBGE, considering the value of January 1^st^, 2022. In addition, the values were converted into US dollars, considering the exchange rate on the same day (US$ 1.00 = R$ 5.63). Six outliers were detected, corresponding to more than three standard deviations from the average, which were corrected by considering the averages of the equivalent semesters in the previous and subsequent years.

The increase in public spending due to the COVID-19 pandemic in 2020 justifies limiting the period analyzed to 2019.

### Statistical Analysis

To analyze the impact of PAF on the outcome variables, an ITS analysis was used by means of autoregressive integrated moving average (ARIMA) modeling in R Studio 4.2.1. The syntax used was based on the work developed by Schaffer et al.^19^

The ITS is a quasi-experimental methodology^15^ ideal for the case under study because, when estimating the effects, the model adjusts to trends that existed prior to the intervention^20^. Considering trends prior to the PAF is essential for assessing the immediate and trend impact of health spending. This technique is widely used in impact assessment and forecasting, and considers factors such as trend, seasonality, and autocorrelation^19^.

Based on the aforementioned definition of PAF^2^ and the results obtained by Chernavsky et al.^1^, 2015 was determined as the date of the intervention. The effects of PAF were estimated using a variable representing the change in level of the outcome variables immediately after its implementation (post-PAF level) and another representing the change in trend post-intervention (post-PAF trend). Significant results, with p < 0.05, were highlighted.

The models were adjusted for autocorrelation of the residuals using the Durbin-Watson test^21^. The best parameters were selected using the auto.arima function from the “astsa” library for R, which takes into account the Bayesian (BIC) and Akaike (AIC) information quality criteria^22^.

The counterfactual forecast was calculated using the “forecast” library and the ARIMA model, considering only the pre-intervention values. To create an indicator of the effects of the PAF, in order to estimate the impact of the final model in relation to the counterfactual forecast, its percentage difference was calculated for the first half of 2015, i.e. immediately after the implementation of the PAF and in the second period of 2019.

Trends prior to the PAF (2010–2014) were estimated using linear regression. The Dickey-Fuller test^23^ was used to assess the level of significance of the trends.

### Ethical Issues

As the data is in the public domain, the study was exempt from ethical review by the research ethics committee.

## RESULTS

Table 1 shows that socio-economic and health system conditions improve as the population size of municipalities increases, except for potential primary care coverage, which is higher in small municipalities. These municipalities have low levels of urbanization, MHDI and life expectancy at birth, as well as few doctors and almost no supplementary healthcare. The larger municipalities have the best socio-economic conditions and the highest number of doctors and population with private health plans, but the lowest potential primary care coverage. Medium-sized municipalities have intermediate results and are close to the national average.

**Table 1:** Sociodemographic and health indicators by population size. Brazil, 2019.

Variables	Brazil	SMA	MED	LAR
Sociodemographics				
% of population	100%	42.60%	22.60%	34.80%
No. of municipalities	5,569	5,245	261	63
Life expectancy at birth^a^	73.94	73.33	75.42	75.52
Human Development Index^a^	0.727	0.66	0.744	0.77
Degree of urbanization^a^	85.30%	62.90%	95.50%	99.30%
Health				
Number of doctors per 1,000 inhabitants^b^	2	0.6	1.7	2.9
Primary Care Coverage^b^	74.20%	100%	70.80%	57.40%
Supplementary health coverage^b^	22.90%	3.50%	21.80%	34.80%

SMA: small (up to 100,000 inhabitants); MED: medium (from 100,001 to 400,000 inhabitants); LAR: large (400,001 or more inhabitants).

Sources: Censo Demográfico e Projeções do Instituto Brasileiro de Geografia e Estatística (IBGE - Demographic Census and Projections of the Brazilian Institute of Geography and Statistic), United Nations Development Program (UNDP-2010); Sistema de Cadastro Nacional de Estabelecimentos de Saúde (SCNES - System for National Registry of Health Establishments); Sistema de Informação de Beneficiários (SIB/ANS/MS - Beneficiary Information System).

^a^ Figures based on UNDP 2010.

^b^ Figures based on June 2019.

Table 2 shows the results of the ITS modeling for the outcomes in the periods before (2010 to 2014) and after (2015 to 2019) the PAF. Before implementation, all groups of municipalities showed an upward trend in all indicators. However, the upward trends in own/state resources in medium-sized and small municipalities, as well as in total expenditure in medium-sized municipalities, were not statistically significant.

**Table 2:** Models for analyzing the impact of the fiscal austerity policy (PAF) on health financing in municipalities using interrupted time series (ITS), by municipal size. Brazil, 2010 to 2019.

Indicator	Baseline			Post-PAF		Percentage difference^a^	
	(2010.1–2014.2)			(2015.1–2019.2)			
	2010.1	2014.2	Trend^b^	Post-PAF level (95%CI)	Post-PAF trend (95%CI)	2015.1	2019.2
All municipalities							
Total expenses	63.73	92.41	2.07*	-6.73*	-1.13	-9,23	-11,55
				(-12.46 to -1.00)	(-2.99 to 0.73)		
Resources transferred by the Union	25.59	32.53	0.66*	-2.04	0.37	-12,14	-11,88
				(-6.66 to 2.56)	(-0.70 to 1.44)		
Own and state resources	38.14	59.88	1.40*	-6.46*	-0.47	-6,69	-16,76
				(-10.43 to -2.48)	(-1.22 to 0.28)		
Small municipalities (up to 100,000 inhabitants)							
Total expenses	63.11	92.43	2.33*	-10.39*	-0.31	-12,89	-12,27
				(-16.20 to -4.59)	(-1.45 to 0.83)		
Resources transferred by the Union	17.83	28.64	0.98*	-2.15	1.01*	-13,1	-7,42
				(-8.60 to 4.29)	(0.06 to 1.97)		
Own and state resources	45.28	63.79	1.33	-2.78	0.5	-10,01	1,6
				(-10.20 to 4.64)	(-0.82 to 1.83)		
Medium-sized municipalities (from 100,001 to 400,000 inhabitants)							
Total expenses	58.83	84.2	1.97	-5.33*	-1.1	-8,14	-11,87
				(-10.00 to -0.67)	(-2.61 to 0.40)		
Resources transferred by the Union	24.78	29.87	0.51*	0.78	-1.1	0,06	-15,84
				(-5.10 to 3.52)	(-4.61 to 2.45)		
Own and state resources	34.05	54.33	1.36	-6.55*	-0.36	-7,61	-16,12
				(-9.35 to -3.76)	(-0.91 to 0.18)		
Large municipalities (more than 400,001 inhabitants)							
Total expenses	69.23	100.61	2.08*	-5.52	-1.54	-7,74	-14,34
				(-11.61 to 0.57)	(-3.51 to 0.43)		
Resources transferred by the Union	34.16	39.07	0.48*	-1.37	-2.53*	-12,35	-27,52
				(-3.74 to 1.00)	(-4.48 to -0.59)		
Own and state resources	35.07	61.54	1.51*	-5.29*	-0.68*	-5,28	-13,38
				(-7.61 to -2.98)	(-1.01 to -0.35)		

Source: Sistema de Informações sobre Orçamentos Públicos em Saúde (SIOPS - Information System on Public Health Budget).

Note: values adjusted by the Special Extended National Consumer Price Index (IPCA-E) for 01/01/2022 and converted into US dollars at the same day’s exchange rate (US$ 1.00 = R$ 5.63).

^a^ Percentage difference between predicted and counterfactual.

^b^ Linear coefficient of the regression in the baseline period. The significance of the trend was tested using the Dickey-Fuller test.

* Considered significant at a 95% confidence level (95%CI).

**Figure 1:**
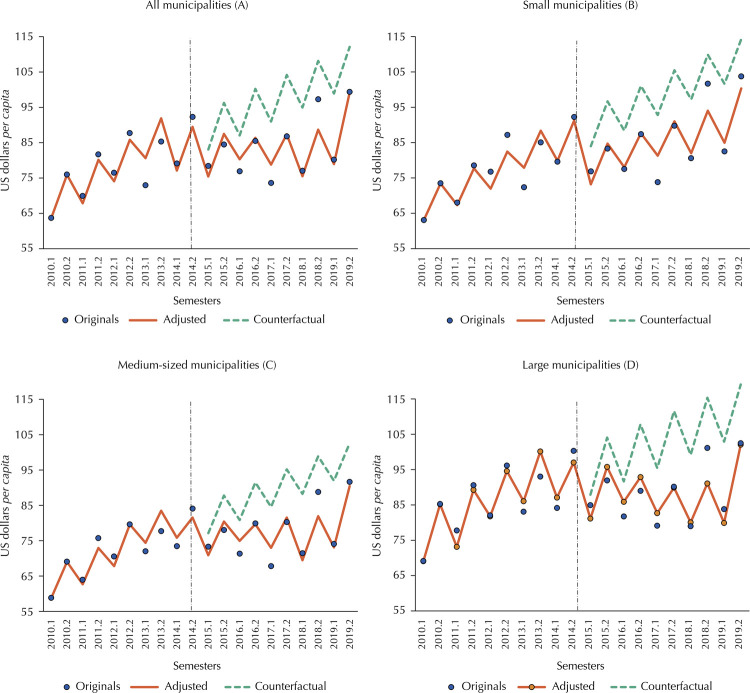
Total six-month *per capita* health expenditure in Brazilian municipalities and interrupted time series models, in current valuesa, in U$$a, by population size, 2010–2019.

Source: *Sistema de Informações sobre Orçamentos Públicos em Saúde* (SIOPS – Information System on Public Health Budget); *Censo e Projeções Populacionais do Instituto Brasileiro de Geografia e Estatística* (IBGE - Demographic Census and Projections from the Brazilian Institute of Geography and Statistics).

Note: (A) All Brazilian municipalities; (B) Small municipalities (up to 100,000 inhabitants); (C) Medium-sized municipalities (100,001 to 400,000 inhabitants); (D) Large municipalities (400,001 or more inhabitants), considering the population in 2019.

^a^ Values adjusted by the Special Extended National Consumer Price Index (IPCA-E) for 01/01/2022 and converted to US dollars at the same day’s exchange rate (US$ 1.00 = R$ 5.63).

Regarding total health expenditure, soon after the PAF began, Brazilian municipalities had on average a significant reduction of US$ 6.70 per inhabitant, with the sharpest drop among small municipalities (US$ 10.39 per inhabitant). Medium-sized and large municipalities had a similar level of decrease, around 5.4 dollars per inhabitant, although the result was only significant for the first group (Table 2 and Figure 1).

**Figure 2:**
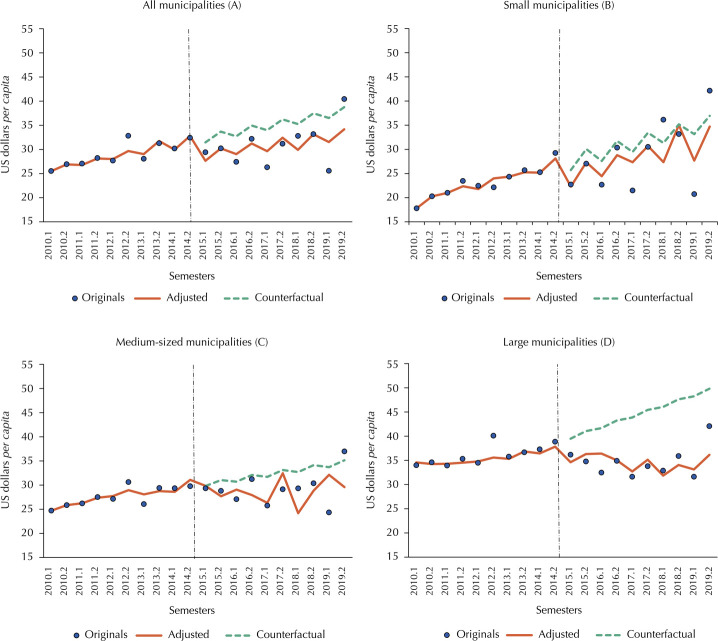
Six-month *per capita* health resources transferred by the Federal Government to Brazilian municipalities and interrupted time series models, in current valuesa, in US$a, by population size, 2010–2019.

Source: *Sistema de Informações sobre Orçamentos Públicos em Saúde* (SIOPS – Information System on Public Health Budget); *Censo e Projeções Populacionais do Instituto Brasileiro de Geografia e Estatística* (IBGE - Demographic Census and Projections from the Brazilian Institute of Geography and Statistics).

Note: (A) All Brazilian municipalities; (B) Small municipalities (up to 100,000 inhabitants); (C) Medium-sized municipalities (100,001 to 400,000 inhabitants); (D) Large municipalities (400,001 or more inhabitants), considering the population of residents in 2019.

^a^ Values adjusted by the Special Extended National Consumer Price Index (IPCA-E) for 01/01/2022 and converted into US dollars at the same day’s exchange rate (US$ 1.00 = R$ 5.63).

**Figure 3:**
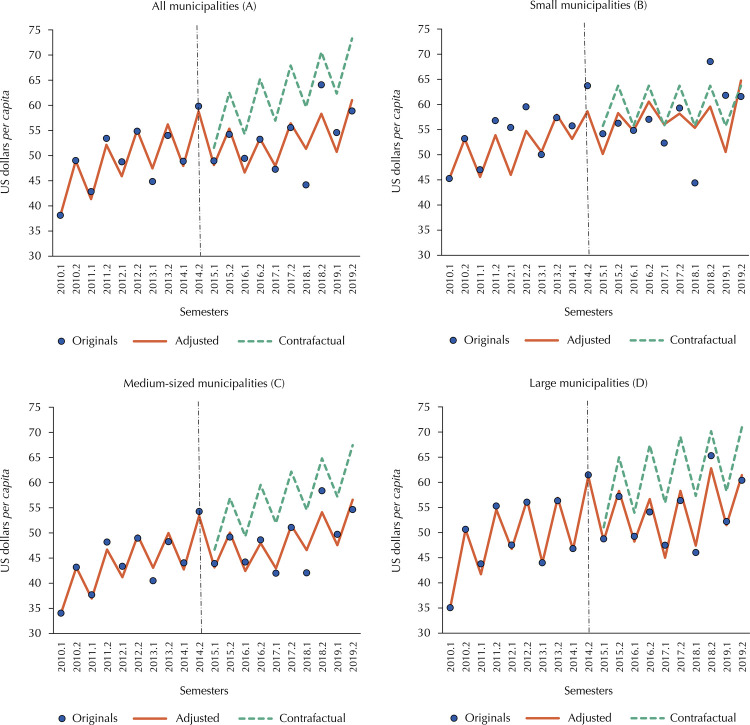
Six-month *per capita* health resources owned or transferred by the states to Brazilian municipalities and interrupted time series models, in current valuesa, in US$a, by population size, 2010–2019.

Source: *Sistema de Informações sobre Orçamentos Públicos em Saúde* (SIOPS – Information System on Public Health Budget); *Censo e Projeções Populacionais do Instituto Brasileiro de Geografia e Estatística* (IBGE - Demographic Census and Projections from the Brazilian Institute of Geography and Statistics).

Note: (A) All Brazilian municipalities; (B) Small municipalities (up to 100,000 inhabitants); (C) Medium-sized municipalities (100,001 to 400,000 inhabitants); (D) Large municipalities (400,001 or more inhabitants), considering the population of residents in 2019.

^a^ Values adjusted by the Special Extended National Consumer Price Index (IPCA-E) for 01/01/2022 and converted to US dollars at the same day’s exchange rate (US$ 1.00 = R$ 5.63).

Concerning resources transferred by the Federal Government, it can be seen that the PAF did not have a significant impact on any group of municipalities in the first half of 2015. For own/state resources, the only group of municipalities that did not see a significant drop was small municipalities. Medium-sized municipalities were the most affected by the immediate loss of this type of resource, closely followed by large municipalities (Table 2 and Figures 2 and 3).

After the implementation of the PAF, no significant changes were observed in the trends of total expenditure, resources transferred by the Union, and own/state resources for the national average and the group of medium-sized municipalities. Small municipalities, on the other hand, showed a trend towards a significant increase in the resources transferred by the Federal Government, on average by US$ 1.00 per inhabitant each semester. Large municipalities, on the other hand, were the most affected in the medium term, with a significant downward trend in their own/state resources and, above all, in the resources transferred by the Federal Government (Table 2).

Comparing the percentage differences between the values predicted by the ARIMA model and the scenarios without the PAF (counterfactuals), it is clear that all groups of municipalities would have experienced an increase in health expenditure, in the resources transferred by the Union, and in their own/state resources (Table 2). Exceptions are observed only in the first half of 2015 for the resources transferred by the Union to medium-sized municipalities and in the second half of 2019 for the own/state resources of small municipalities.

Figure 1 visually shows the ARIMA model for total health expenditure and counterfactuals. None of the municipal groups would have reached the level of expenditure predicted by the counterfactual model at the end of the period analyzed. In other words, in the absence of the intervention, all groups of municipalities would have been allocating a greater volume of resources to health throughout the period following the PAF.

Figure 2 shows the results of the ITS models for the resources transferred by the Federal Government. Over the period analyzed, medium-sized municipalities almost stagnated in terms of the volume of resources, leaving them at a disadvantage compared to the other population sizes at the end of the time series. Small municipalities and the national average decreased in 2015, but then recovered and came close to the counterfactual. As for the larger municipalities, there was a reversal in the trend of these resources after the implementation of the PAF, generating a gap between the values predicted by the model and the expected scenario.

About own/state resources earmarked for health, we can see that, except for small municipalities, the graphs show similar behavior (Figure 3). Among large and medium-sized municipalities, there was a decrease in this type of funding from 2015 onwards, apparently accentuated in subsequent semesters, followed by a slight recovery from 2017 onwards. On the other hand, small municipalities had already been suffering stagnation in the volume of own/state resources before 2015, as indicated by the counterfactual model.

## DISCUSSION

On the national average, the results of the model show that the implementation of the PAF had an immediate negative impact on total health expenditure and on municipalities’ own/state resources. This impact was not uniform between the different groups, with small municipalities experiencing significant drops only in total expenditure, large ones only in own/state resources, and medium-sized ones experiencing a decline in both variables, while there was no reduction in the resources transferred by the Union in any of these.

In the medium-term, the implementation of the PAF only had a negative impact on large municipalities, which saw significant reductions in their own/state and federally transferred resources.

Some studies^4,5^, when verifying the decrease in health expenditure in municipalities from 2015 onwards, attribute responsibility to the economic crisis and the resulting drop in revenue. However, international experience^24,25^ shows that economic crises do not necessarily have a negative impact on the financing of social policies. It is important to emphasize that the implementation of the PAF was a choice and not a natural and immediate consequence of the crisis.

From an epidemiological and public health perspective, cutting social spending in a context of crisis means increasing the negative effects on the population’s health: it increases risks (e.g. alcohol and tobacco abuse, malnutrition, and environmental risks); decreases access to promotion, prevention, and care services; and increases morbidity and mortality^6^.

The discrepancy in funding between different sizes of municipality validates the methodological approach of this study. The literature mentions the link between population size and the allocation of health resources^5,26^. In general, medium-sized municipalities spend less on health with their own resources than small and large ones^26^. Between 2003 and 2019, large municipalities spent more *per capita* on resources from transfers^5^. However, as indicated by Cruz et al.^5^ and this study, small municipalities had the highest growth in *per capita* expenditure with transferred resources in the period.

Total health expenditure in small municipalities suffered the greatest impact immediately after the PAF. Cruz et al.^5^ suggest that the general drop in the level of health expenditure in Brazilian municipalities in 2015 was predominantly due to a decrease in municipal revenue. Although the indicators in this study do not make it possible to single out expenditure from own resources, the fact is that small municipalities are more dependent on external resources to finance health actions^5^, which is natural, since municipal revenues are largely linked to the provision of services and the circulation of goods^27^, which are lower in these places. Thus, an in-depth analysis of the behavior of health expenditure in municipalities of this size in crisis situations is necessary in future studies.

Also regarding small municipalities, there was an unexpected increase in the trend of resources transferred by the Federal Government post-PAF. It should be noted, however, that large municipalities showed a downward trend in this indicator, indicating a redistribution rather than an increase in the resources provided. This shows that, to a certain extent, the Federal Government’s role of redistributing fiscal capacity and the provision of public services throughout the territory^28^ has been exercised, since small municipalities had lower *per capita* expenditure on health (Figure 1).

The results for the medium-sized municipalities are similar to those of the national average: an immediate reduction in total expenditure and in own/state resources, with no change in post-PAF trends in all the variables studied. The heterogeneity of this group of municipalities in terms of health system structure and demographic and economic characteristics^14^ makes it difficult to interpret these results. Without losing sight of this, it should be noted that they had the lowest *per capita* expenditure over the entire period. In other words, although they were not the most impacted by the PAF, these are the municipalities most in need of resources. The lack of impact only suggests that the problem predates the PAF.

Regarding large municipalities, it was shown that total health expenditure after the PAF only reached 2015 levels, around US$ 185 dollars *per capita* per year, in 2019. It is therefore reasonable to propose that the greatest impact of the PAF was on this group, since small and medium-sized municipalities recovered their spending levels a year earlier.

The fall in the trend of funds transferred by the Federal Government has the largest share in the losses of large municipalities. The increase in the volume of funds allocated by parliamentary amendments (EP) to health from 2016 onwards partly explains this change. According to Piola and Vieira^29^, while 1.7% of total federal spending on health in 2015 came from EP, this percentage has exceeded 7% since 2016. Around 75% of these funds were transferred to states and municipalities, especially small ones, due to the preference of parliamentarians to allocate funds to places closer to the beneficiary population^29,30^.

Although this allocation is partly desirable, since these municipalities are more vulnerable (Table 1), the distribution of these resources is not equitable between municipalities, which can lead to inequality^30^. In addition, the increase in the share of spending allocated by EP, together with the freezing of health spending in the federal budget, means a reduction in the share of own allocation by the Ministry of Health^29^, which implies a decrease in its inducing power^31^. In addition, studies have highlighted a lack of budgetary transparency in the resources allocated by the EP^29,30^.

Except for large municipalities, the resources transferred by the Federal Government were less affected by the implementation of the PAF. A previous study^12^ showed that the percentage of federal health resources transferred directly to municipalities has remained relatively stable since 2004, although the *per capita* amounts have increased. In this sense, the importance of rules that establish regular funding for health services,such as the Primary Care Floor, should be emphasized. The considerable volume of revenue from these rules^12^ has helped to soften the impact of the PAF on health financing.

Future studies will need to investigate which sectors of municipal services were most affected, considering the demographic particularities of each municipality. It is known that the implementation of the PAF has influenced the loss of quality in oral health services^3^. The literature also mentions a decrease in access to medicines provided by the SUS due to reduced spending at municipal and state level in 2015 and 2016^32^.

An issue indirectly raised by the results concerns the fiscal dependence of municipalities in relation to health spending. Small and medium-sized municipalities experienced a decrease in spending on their own/state resources, in relation to resources from the Federal Government, indicating a growing dependence on federal resources for health spending. Cruz et al.^5^ when analyzing municipal revenues, observed a significant increase in non-own revenues in relation to own revenues from 2016 onwards, especially among small municipalities, which suggests that the growth in fiscal dependence is not limited to the health area.

The availability of resources for municipalities is essential for decentralizing the SUS and strengthening PHC. In recent decades, the increase in public resources allocated to health has been due to the growth in municipal spending, sustained mainly by the allocation of own resources^12^. However, the results of this study show that these resources are susceptible to economic cycles and the political decisions of the Federal Government. This corroborates the idea that municipalities lack a more regular and stable source of tax revenue^27^.

Regarding the tax collection capacity of Brazilian municipalities, it should also be noted that, while this article is being written, the Constitution Amendment Bill No. 45 of 2019, which proposes changes to taxation mechanisms in all spheres of the Brazilian state,is being processed.

The results of this article should be interpreted with caution. The ITS models showed that the implementation of the PAF had no statistically significant impact on total health expenditure in the medium term in the municipal groups analyzed, although a slight decline occurred in all of them. At best, it can be inferred that these expenses have stagnated since the PAF.

It should be borne in mind that Brazil’s public spending on health is low compared to countries with similar incomes that don’t have universal systems^13^. Argentina and Chile, for example, in 2015 had higher public spending as a percentage of gross domestic product (around 1%) and *per capita* (40% and 50% higher, respectively)^13^. Contrasting reality with the ambitious principles of the SUS, it becomes clear that agendas of cuts or even stagnation in public spending are incompatible with the effective realization of our health system^13^.

An important limitation of this study stems from the decision not to use the indicator “Share of transfers to health in relation to the municipality’s total health expenditure”, made available by SIOPS. This choice was made due to changes in the coding of income and expenditure for the indicator in 2016, 2017, and 2019, as explained by the Coordination of the Information System on Public Health Budget (CSIOPS/CGES/DESID/SE/MS), in response to the authors’ inquiry. These changes make it difficult to carry out a precise longitudinal analysis of the states’ participation and their own spending on health in the municipalities.

Other important limitations should be highlighted. The first stems from the quality of the data provided by SIOPS, which has been criticized for its reliability and completeness^33^. In this study, the incompleteness of the data was a characteristic that was present, especially in small municipalities, which may have overestimated their expenditure. A final limitation concerns the size of the time series analyzed. The small number of points in time makes it difficult to identify significant trends or seasonal patterns, to properly estimate the autoregression (AR) and moving average (MA) parameters, as well as increasing the sensitivity of the models to outliers^19^.

On the other hand, the study stands out for its nationwide estimation of the impact of the PAF on municipal health financing based on ITS, a quasi-experimental method suitable for studies evaluating the impact of public policies^19^.

Although the PAF has been interrupted because of the pandemic, it still occupies an important place on the public agenda. Brazil from 2015 to 2019 has become a social laboratory, providing an opportunity to evaluate and denounce the consequences of austerity on the SUS and the health of the population. The role of fiscal policy in stabilizing the economy cannot serve as an argument for suppressing health spending, given that it has redistributive and multiplier effects^34^. As Ocké-Reis^34^ (2023) points out, the creation of a new fiscal framework should consider the need to increase public spending on health to at least 6% of gross domestic product, with a view to bringing the assumptions of the SUS closer to reality.
