# Supplementary material for: Fiscal austerity and municipal health spending: an interrupted time series study
Source: Rev Saude Publica. 2024 Sep 16;58:42. doi: 10.11606/s1518-8787.2024058005772 (PMC11548907; doi:10.11606/s1518-8787.2024058005772)
Supplement: Translation

# Translation: Austeridade fiscal e gastos municipais em saúde: estudo de séries temporais interrompidas

**Authors:** Leo Ramos Maia, Mônica Rodrigues Campos, Débora Castanheira

**Affiliations:** I Brasil Fundação Oswaldo Cruz. Escola Nacional de Saúde Pública Sergio Arouca. Programa de Pós-Graduação em Saúde Pública. Rio de Janeiro, Brasil; II Brasil Fundação Oswaldo Cruz. Escola Nacional de Saúde Pública Sergio Arouca. Departamento de Ciências Sociais. Rio de Janeiro, Brasil; III Brasil Fundação Oswaldo Cruz. Instituto Nacional de Infectologia Evandro Chagas. Rio de Janeiro, Brasil

**Keywords:** Financiamento dos Sistemas de Saúde, Política Fiscal, Gastos Públicos com Saúde, Cidades

## Abstract

**OBJETIVO:**

Analisar o impacto da política de austeridade fiscal (PAF) nas despesas em saúde dos municípios brasileiros, levando em consideração o porte populacional e a fonte dos recursos.

**MÉTODOS:**

Utilizou-se o método de séries temporais interrompidas para analisar o efeito da PAF sobre as despesas totais, recursos transferidos pela União e recursos próprios/estaduais *per capita* destinados à saúde nos municípios. A série temporal analisada compreendeu o período de 2010 a 2019, com periodicidade semestral. Adotou-se o primeiro semestre de 2015 como data de início da intervenção. Os municípios foram agregados em pequenos (até 100 mil habitantes), médios (101 mil a 400 mil habitantes) e grandes (mais de 400 mil habitantes). Os dados foram obtidos no Sistema de Informações sobre Orçamentos Públicos em Saúde.

**RESULTADOS:**

Os resultados para a média nacional dos municípios demonstram que a PAF teve um impacto negativo sobre o nível de despesas totais e de recursos próprios/estaduais destinados à saúde no primeiro semestre de 2015, não provocando mudanças estaticamente significativas nas tendências de nenhum dos indicadores analisados no período posterior a 2015. Municípios pequenos tiveram queda nas despesas totais, enquanto os grandes, nos recursos próprios/estaduais e os médios, em ambas as variáveis. Não houve queda estatisticamente significante no volume de recursos transferidos pela União no momento imediato à implementação da PAF em nenhum dos grupos municipais analisados. No médio prazo, a PAF gerou impacto negativo apenas nos grandes municípios, que tiveram reduções significativas nas tendências de recursos próprios/estaduais e transferidos pela União destinados à saúde.

**CONCLUSÃO:**

No geral, o impacto da PAF no financiamento da saúde dos municípios se deu de forma imediata e a partir da queda de recursos próprios/estaduais destinados à saúde. Nos municípios grandes, entretanto, o impacto foi perdurável entre 2015 e 2019, afetando, principalmente, as despesas com saúde oriundas de recursos da União.

## INTRODUÇÃO

Em 2015 o Brasil iniciou a implementação de uma política de austeridade fiscal (PAF)^1^, definida como uma redução de gastos públicos em anos subsequentes^2^. Sua posterior constitucionalização, a partir da Emenda Constitucional n^o^ 95, de 2016, influenciou a elaboração de diversos estudos voltados aos seus efeitos nas condições de saúde da população e na provisão de serviços e capacidade de financiamento do Sistema Único de Saúde (SUS)^3^.

Os gastos públicos *per capita* destinados à saúde no Brasil, que apresentavam tendência crescente desde 2003, tiveram um inédito decréscimo em 2015^8^, data que marcou o início da implementação da PAF. Posteriormente, verificou-se estagnação das despesas públicas em saúde até 2019^9^, quando, por conta da pandemia de covid-19, decidiu-se romper o teto de gastos e adotar uma política fiscal expansiva^10^.

Cortar gastos públicos em saúde implica reduzir a qualidade e quantidade dos bens e serviços de saúde ofertados para a população brasileira^6^, realidade que penaliza especialmente a população mais pobre, dependente da oferta pública de serviços.

Os estudos que avaliam os efeitos da PAF no financiamento do SUS voltam-se principalmente aos gastos públicos totais e/ou federais^7,9,11^. Acerca das despesas municipais em saúde, sabe-se apenas que, na média nacional, cresceram 124% entre 2004 e 2014, tiveram uma queda de 9% em 2015 e foram gradualmente recuperadas entre 2015 e 2019 (14%)^5^.

É importante analisar o impacto da PAF nos gastos municipais em saúde porque o pacto constitucional em torno da descentralização situou nos municípios grande parte das atribuições do SUS, sendo a principal delas a Atenção Primária à Saúde (APS), que é modelo de gestão nacional^12^. Além disso, o referido crescimento das despesas públicas totais em saúde no período anterior à PAF ocorreu devido ao aumento da participação municipal no financiamento do SUS^10^, dinâmica influenciada pela Emenda Constitucional n^o^ 29 de 2000^13^.

Deve-se considerar ainda que a origem de recursos e o volume das despesas municipais com saúde são desiguais de acordo com o porte municipal^5^, porque as faixas populacionais encontram-se associadas à capacidade de arrecadação, de captação de recursos humanos e à infraestrutura do sistema de saúde^14^, fatores que influenciam na capacidade e composição dos gastos^5^. Análises detalhadas nesse nível de desagregação configuram uma lacuna na literatura.

Nesse sentido, este artigo tem por objetivo analisar o impacto da PAF nas despesas em saúde dos municípios brasileiros, levando em consideração o porte populacional e a fonte dos recursos.

## MÉTODOS

### Desenho e Fontes

Estudo retrospectivo, quantitativo e analítico que utilizou o método de regressão de dados de séries temporais interrompidas (ITS – *Interrupted Time Series*)^15^. Utilizou-se como fonte o Sistema de Informações sobre Orçamentos Públicos em Saúde (SIOPS)^16^, além de dados populacionais provenientes do censo demográfico (2010) e projeções, do Instituto Brasileiro de Geografia e Estatística (IBGE).

Os dados da plataforma do SIOPS não estão disponíveis em formato de banco de dados^16^, por esse motivo os dados utilizados nesta análise foram solicitados via Lei de Acesso à Informação. Verificou-se a existência de incompletude nos dados (média de 10% no período), especialmente no período de 2010 a 2013.

Os municípios foram agregados em três portes populacionais: pequenos (até 100.000 habitantes), médios (de 100.001 a 400.000 habitantes) e grandes (400.001 ou mais habitantes), considerando os residentes em 2019. Brasília não foi incluída na análise, pois suas fontes de recursos são distintas das municipais.

Para a caracterização de cada grupo, foram selecionados indicadores de aspectos socioeconômicos e do sistema de saúde: 1) Esperança de vida ao nascer; 2) Índice de Desenvolvimento Humano Municipal (IDHM); 3) Grau de urbanização; 4) Número de médicos por 1.000 habitantes; 5) Cobertura potencial da atenção básica^17^; e 6) Cobertura da saúde complementar, que expressa a porcentagem da população beneficiária de planos privados de saúde.

Os indicadores são oriundos das seguintes fontes: (1), (2) e (3) – estimativas do Programa das Nações Unidas para o Desenvolvimento (PNUD) com base no censo demográfico de 2010; (4) – Sistema de Cadastro Nacional de Estabelecimentos de Saúde (SCNES) e projeções populacionais do IBGE; (5) – Sistema de Informação e Gestão da Atenção Básica (e-Gestor AB); e (6) – Agência Nacional de Saúde Suplementar (ANS).

### Indicadores de Desfecho

Foram estimados três indicadores relacionados ao financiamento da saúde de 2010 a 2019, com periodicidade semestral:

1. Despesas totais com saúde: representa a despesa liquidada total com saúde (exceto aposentadorias e pensões), inclusive aquela financiada por outras esferas de governo, por habitante^18^.
2. Recursos transferidos pela União: representa o total de receitas realizadas em saúde transferidas pela União, por habitante.
3. Recursos próprios e estaduais: representa o total de despesas liquidadas com saúde (exceto aposentarias e pensões), subtraído os recursos transferidos pela União, por habitante.

O nível nacional dos indicadores de desfecho foi estimado a partir da média aritmética dos três grupos de municípios.

Todos os valores foram ajustados pela perda inflacionária a partir do Índice Nacional de Preços ao Consumidor Especial (IPCA-E), fornecido pelo IBGE, considerando o valor de 1^o^ de janeiro de 2022. Além disso, os valores foram convertidos para dólares americanos, considerando a cotação do mesmo dia (US$1,00 = R$5,63). Detectou-se a presença de seis *outliers*, correspondentes a mais de três desvios-padrões em relação à média, que foram corrigidos considerando as médias dos semestres equivalentes nos anos anterior e posterior.

O aumento dos gastos públicos em decorrência da pandemia da covid-19 em 2020 justifica a delimitação do período analisado até 2019.

### Análise Estatística

Para analisar o impacto da PAF nas variáveis de desfecho, foi empregada uma análise de ITS por meio de modelagem autorregressiva integrada de médias móveis (ARIMA) no R Studio 4.2.1. A sintaxe utilizada foi baseada no trabalho desenvolvido por Schaffer et al.^19^

O ITS é metodologia quasi-experimental^15^ ideal para o caso em estudo porque, ao estimar os efeitos, o modelo se ajusta às tendências preexistentes à intervenção^20^. Considerar tendências anteriores à PAF é essencial para avaliar o impacto imediato e na tendência dos gastos em saúde. Tal técnica é amplamente utilizada na avaliação do impacto e previsões, e considera fatores como tendência, sazonalidade e autocorrelação^19^.

Com base na definição supracitada de PAF^2^ e nos resultados obtidos por Chernavsky et al.^1^, determinou-se 2015 como a data da intervenção. Os efeitos da PAF foram estimados utilizando uma variável representando a mudança de nível das variáveis de desfecho imediatamente após sua implementação (nível pós-PAF) e outra representando a mudança de tendência pós-intervenção (tendência pós-PAF). Resultados significativos, com p < 0,05, foram destacados.

Os modelos foram ajustados para autocorrelação dos resíduos utilizando o teste de Durbin-Watson^21^. Os melhores parâmetros foram selecionados utilizando a função auto.arima da biblioteca “astsa” para R, que leva em consideração os critérios de qualidade de informação Bayesiano (BIC) e de Akaike (AIC)^22^.

A previsão contrafactual foi calculada utilizando a biblioteca “*forecast*” e o modelo ARIMA, considerando apenas os valores pré-intervenção. Para criar um indicador dos efeitos da PAF, de forma a estimar o impacto do modelo final em relação à previsão contrafactual, calculou-se sua diferença percentual para o primeiro semestre de 2015, ou seja, imediatamente após a implementação da PAF e no segundo período de 2019.

As tendências anteriores à PAF (2010–2014) foram estimadas por meio de regressão linear. O teste Dickey-Fuller^23^ foi realizado para aferir o nível de significância das tendências.

### Questões Éticas

Por se tratar de dados de domínio público, o estudo foi dispensado de apreciação ética pelo comitê de ética em pesquisa.

## RESULTADOS

A Tabela 1 mostra que as condições socioeconômicas e do sistema de saúde melhoram com o aumento do porte populacional dos municípios, exceto a cobertura potencial da atenção básica, que é maior nos municípios pequenos. Esses municípios têm baixo grau de urbanização, IDHM e esperança de vida ao nascer, além de poucos médicos e quase nenhuma saúde suplementar. Os municípios grandes têm as melhores condições socioeconômicas e o maior número de médicos e de população com planos privados de saúde, mas a menor cobertura potencial da atenção básica. Os municípios médios têm resultados intermediários e próximos à média nacional.

**Tabela 1:** Indicadores sociodemográficos e de saúde segundo porte populacional. Brasil, 2019.

Variáveis	Brasil	PEQ	MED	GRA
Sociodemográficos				
% da população	100%	42,60%	22,60%	34,80%
Nº de municípios	5.569	5.245	261	63
Esperança de vida ao nascer^a^	73,94	73,33	75,42	75,52
Índice de Desenvolvimento Humano^a^	0,727	0,66	0,744	0,77
Grau de urbanização^a^	85,30%	62,90%	95,50%	99,30%
Saúde				
Nº de médicos por 1.000 habitantes^b^	2	0,6	1,7	2,9
Cobertura atenção básica^b^	74,20%	100%	70,80%	57,40%
Cobertura saúde suplementar^b^	22,90%	3,50%	21,80%	34,80%

PEQ: pequenos (até 100.000 habitantes); MED: médios (de 100.001 a 400.000 habitantes); GRA: grandes (400.001 ou mais habitantes).

Fontes: Censo Demográfico e Projeções do Instituto Brasileiro de Geografia e Estatística (IBGE), Programa das Nações Unidas para o Desenvolvimento (PNUD - 2010), Sistema de Cadastro Nacional de Estabelecimentos de Saúde (SCNES) e Sistema de Informação de Beneficiários (SIB/ANS/MS).

^a^ Valores com base em PNUD 2010.

^b^ Valores com base em junho de 2019.

A Tabela 2 apresenta os resultados da modelagem ITS para os desfechos, nos períodos anteriores (2010 a 2014) e posteriores (2015 a 2019) à PAF. Antes da implementação, todos os grupos de municípios apresentavam tendência de crescimento em todos os indicadores. No entanto, as tendências de aumento nos recursos próprios/estaduais nos municípios de médio e pequeno porte, assim como nas despesas totais nos municípios de médio porte, não foram significativas estatisticamente.

**Tabela 2:** Modelos de análise do impacto da política de austeridade fiscal (PAF) sobre o financiamento da saúde nos municípios por meio de séries temporais interrompidas, segundo porte municipal. Brasil, 2010 a 2019.

Indicador	Baseline			Pós-PAF		Diferença percentual^a^	
	(2010.1–2014.2)			(2015.1–2019.2)			
	2010.1	2014.2	Tendência^b^	Nível pós-PAF (IC95%)	Tendência pós-PAF (IC95%)	2015.1	2019.2
Todos os municípios							
Despesas totais	63,73	92,41	2,07*	-6,73*	-1,13	-9,23	-11,55
				(-12,46 a -1,00)	(-2,99 a 0,73)		
Recursos transferidos pela União	25,59	32,53	0,66*	-2,04	0,37	-12,14	-11,88
				(-6,66 a 2,56)	(-0,70 a 1,44)		
Recursos próprios e estaduais	38,14	59,88	1,40*	-6,46*	-0,47	-6,69	-16,76
				(-10,43 a -2,48)	(-1,22 a 0,28)		
Municípios pequenos (até 100.000 residentes)							
Despesas totais	63,11	92,43	2,33*	-10,39*	-0,31	-12,89	-12,27
				(-16,20 a -4,59)	(-1,45 a 0,83)		
Recursos transferidos pela União	17,83	28,64	0,98*	-2,15	1,01*	-13,1	-7,42
				(-8,60 a 4,29)	(0,06 a 1,97)		
Recursos próprios e estaduais	45,28	63,79	1,33	-2,78	0,5	-10,01	1,6
				(-10,20 a 4,64)	(-0,82 a 1,83)		
Municípios médios (de 100.001 até 400.000 residentes)							
Despesas totais	58,83	84,2	1,97	-5,33*	-1,1	-8,14	-11,87
				(-10,00 a -0,67)	(-2,61 a 0,40)		
Recursos transferidos pela União	24,78	29,87	0,51*	0,78	-1,1	0,06	-15,84
				(-5,10 a 3,52)	(-4,61 a 2,45)		
Recursos próprios e estaduais	34,05	54,33	1,36	-6,55*	-0,36	-7,61	-16,12
				(-9,35 a -3,76)	(-0,91 a 0,18)		
Municípios grandes (mais de 400.001 residentes)							
Despesas totais	69,23	100,61	2,08*	-5,52	-1,54	-7,74	-14,34
				(-11,61 a 0,57)	(-3,51 a 0,43)		
Recursos transferidos pela União	34,16	39,07	0,48*	-1,37	-2,53*	-12,35	-27,52
				(-3,74 a 1,00)	(-4,48 a -0,59)		
Recursos próprios e estaduais	35,07	61,54	1,51*	-5,29*	-0,68*	-5,28	-13,38
				(-7,61 a -2,98)	(-1,01 a -0,35)		

Fonte: Sistema de Informações sobre Orçamentos Públicos em Saúde (SIOPS).

Nota: valores ajustados pelo Índice Nacional de Preços ao Consumidor Especial (IPCA-E) para 01/01/2022 e convertidos para dólares americanos da cotação do mesmo dia (US$1,00 = R$5,63).

^a^ Diferença percentual entre o predito e o contrafacto.

^b^ Coeficiente linear da regressão no período baseline. A significância da tendência foi testada por meio do teste Dickey-Fuller.

* Considerado significativo a um nível de confiança de 95% (IC95%).

No que tange às despesas totais em saúde, logo após o início da PAF, os municípios brasileiros tiveram, em média, uma redução significativa de US$ 6,70 por habitante, com queda mais acentuada entre os municípios de pequeno porte (US$ 10,39 por habitante). Municípios de médio e grande porte tiveram um nível de perda similar, por volta de 5,4 dólares/habitante, embora o resultado só tenha se mostrado significativo para o primeiro grupo (Tabela 2 e Figura 1).

**Figura 1:** Despesas totais semestrais *per capita* em saúde nos municípios brasileiros e modelos de séries temporais interrompidas, em valores correntesa, em US$a, por porte populacional, 2010–2019.

Fonte: Sistema de Informações sobre Orçamentos Públicos em Saúde (SIOPS); Censo e Projeções Populacionais do Instituto Brasileiro de Geografia e Estatística (IBGE).

(A) Todos os municípios brasileiros. (B) Pequeno porte (até 100.000 habitantes). (C) médio porte (de 100.001 a 400.000 habitantes). (D) Grande porte (400.001 ou mais habitantes), considerando a população de residentes no ano de 2019.

^a^ Valores ajustados pelo Índice Nacional de Preços ao Consumidor Especial (IPCA-E) para 01/01/2022 e convertidos para dólares americanos da cotação do mesmo dia (US$1,00 = R$5,63).

Com relação aos recursos transferidos pela União, observa-se que a PAF não impactou significativamente nenhum grupo de municípios no 1º semestre de 2015. Para os recursos próprios/estaduais, o único grupo de municípios que não teve queda significativa foi o de pequeno porte. Municípios médios foram os mais afetados pela perda imediata desse tipo de recursos, seguidos de perto pelos de grande porte (Tabela 2 e Figuras 2 e 3).

Após a implantação da PAF, não foram observadas mudanças significativas nas tendências de despesas totais, recursos transferidos pela União e recursos próprios/estaduais para a média nacional e no grupo de municípios de médio porte. Municípios de pequeno porte, por sua vez, apresentaram tendência de aumento significativa dos recursos transferidos pela União, em média de US$ 1,00 por habitante a cada semestre. Já os municípios de grande porte foram os mais afetados no médio prazo, com uma diminuição de tendência significativa nos recursos próprios/estaduais e, principalmente, nos recursos transferidos pela União (Tabela 2).

Comparando as diferenças percentuais entre os valores preditos pelo modelo ARIMA e os cenários sem a PAF (contrafactuais), fica claro que todos os grupos de municípios teriam experimentado um aumento em despesas de saúde, nos recursos transferidos pela União e nos próprios/estaduais (Tabela 2). Exceções são observadas apenas no primeiro semestre de 2015 para os recursos transferidos pela União para municípios médios e no segundo semestre de 2019 para os recursos próprios/estaduais de municípios pequenos.

A Figura 1 apresenta visualmente o modelo ARIMA para despesas totais em saúde e contrafactos. Nenhum dos grupos municipais alcançaria o nível de despesas previstos pelo modelo contrafactual no final do período analisado. Ou seja, na ausência da intervenção, todos os grupos de municípios estariam destinando maior volume de recursos para a saúde em todo o período posterior à PAF.

A Figura 2 mostra os resultados dos modelos de ITS para os recursos transferidos pela União. No período analisado, os municípios médios quase estagnaram em volume de recursos, ficando em desvantagem em relação aos demais portes populacionais ao final da série histórica. Os municípios pequenos e a média nacional tiveram uma queda em 2015, mas depois se recuperaram e se aproximaram do contrafactual. Já nos municípios grandes, houve inversão na tendência desses recursos após a implementação da PAF, gerando um descolamento entre os valores previstos pelo modelo e o cenário esperado.

**Figura 2:** Recursos semestrais *per capita* em saúde transferidos pela União para os municípios brasileiros e modelos de séries temporais interrompidas, em valores correntesa, em US$a, por porte populacional, 2010–2019.

Fonte: Sistema de Informações sobre Orçamentos Públicos em Saúde (SIOPS); Censo Populacional do Instituto Brasileiro de Geografia e Estatística (IBGE).

Nota: (A) Todos os municípios brasileiros; (B) Pequeno porte (até 100.000 habitantes); (C) Médio porte (de 100.001 a 400.000 habitantes); (D) Grande porte (400.001 ou mais habitantes), considerando a população de residentes no ano de 2019.

^a^ Valores ajustados pelo Índice Nacional de Preços ao Consumidor Especial (IPCA-E) para 01/01/2022 e convertidos para dólares americanos da cotação do mesmo dia (US$1,00 = R$5,63).

Quanto aos recursos próprios/estaduais destinados à saúde podemos observar que, à exceção dos municípios pequenos, os gráficos possuem comportamentos semelhantes (Figura 3). Entre municípios grandes e médios, nota-se uma queda desse tipo de recursos a partir de 2015, aparentemente acentuada nos semestres subsequentes, seguida de uma leve recuperação a partir de 2017. Por sua vez, municípios de pequeno porte já vinham sofrendo estagnação no volume de recursos próprios/estaduais antes de 2015, conforme indicado pelo modelo contrafactual.

**Figura 3:** Recursos semestrais *per capita* em saúde próprios ou transferidos pelos estados para os municípios brasileiros e modelos de séries temporais interrompidas, em valores correntesa, em US$a, por porte populacional, 2010–2019.

Fonte: Sistema de Informações sobre Orçamentos Públicos em Saúde (SIOPS); Censo e Projeções Populacionais do Instituto Brasileiro de Geografia e Estatística (IBGE).

Nota: (A) Todos os municípios brasileiros; (B) Pequeno porte (até 100.000 habitantes); (C) Médio porte (de 100.001 a 400.000 habitantes); (D) Grande porte (400.001 ou mais habitantes), considerando a população de residentes no ano de 2019.

^a^ Valores ajustados pelo Índice Nacional de Preços ao Consumidor Especial (IPCA-E) para 01/01/2022 e convertidos para dólares americanos da cotação do mesmo dia (US$1,00 = R$5,63).

## DISCUSSÃO

Na média nacional, os resultados do modelo demonstram que a implementação da PAF teve um impacto negativo imediato nas despesas totais em saúde e nos recursos próprios/estaduais dos municípios. Esse impacto não foi uniforme entre os diferentes grupos, municípios pequenos tiveram quedas significativas apenas nas despesas totais, grandes apenas nos recursos próprios/estaduais e médios experimentaram declínio em ambas as variáveis, não havendo redução nos recursos transferidos pela União em nenhum desses.

No médio prazo, a implementação da PAF impactou negativamente apenas os municípios de grande porte, que apresentaram reduções significativas nas tendências de recursos próprios/estaduais e transferidos pela União.

Alguns estudos^4,5^, ao verificarem a diminuição das despesas em saúde nos municípios a partir de 2015, atribuem responsabilidade à crise econômica e à queda de receitas que resultou dela. No entanto, experiências internacionais^24,25^ demonstram que crises econômicas não necessariamente geram impactos negativos sobre o financiamento das políticas sociais. É importante ressaltar que a implementação da PAF foi uma escolha e não uma consequência natural e imediata da crise.

Na perspectiva epidemiológica e de saúde pública, cortar gastos sociais em contexto de crise significa potencializar os efeitos negativos na saúde da população: aumenta riscos (por exemplo: consumo abusivo de álcool e tabaco, desnutrição e riscos ambientais); diminui o acesso aos serviços de promoção, prevenção e cuidado; e eleva a morbimortalidade^6^.

A discrepância no financiamento entre diferentes tamanhos de municípios valida a abordagem metodológica deste estudo. A literatura menciona a ligação entre tamanho populacional e alocação de recursos de saúde^5,26^. Em geral, municípios médios gastam menos em saúde com recursos próprios do que os pequenos e grandes^26^. Entre 2003 e 2019, municípios grandes tiveram maior gasto *per capita* com recursos advindos de transferências^5^. No entanto, como indicado por Cruz et al.^5^ e este estudo, municípios de pequeno porte tiveram o maior crescimento de despesas *per capita* com recursos transferidos no período.

As despesas totais em saúde nos municípios pequenos sofreram maior impacto imediatamente após a PAF. Cruz et al.^5^ sugerem que a queda geral no nível de despesas em saúde nos municípios brasileiros em 2015 ocorreu, predominantemente, pela diminuição das receitas municipais. Apesar de os indicadores desta pesquisa não possibilitarem singularizar despesas oriundas de recursos próprios, o fato é que municípios pequenos são mais dependentes de recursos externos para financiamento das ações de saúde^5^, o que é natural, pois as receitas municipais são em grande parte atreladas à prestação de serviços e à circulação de mercadorias^27^, que são menores nesses locais. Assim, uma análise aprofundada do comportamento das despesas em saúde de municípios desse porte em situações de crise se mostra necessária em estudos futuros.

Ainda sobre municípios pequenos, verificou-se um inesperado crescimento na tendência dos recursos transferidos pela União pós-PAF. Ressalte-se, entretanto, que municípios grandes apresentaram tendência de queda nesse indicador, indicando uma redistribuição em vez de aumento dos recursos aportados. Isso mostra que, em certa medida, a atribuição da União de redistribuir a capacidade fiscal e a oferta dos serviços públicos ao longo do território^28^ tem sido exercida, já que municípios pequenos tinham menor gasto *per capita* em saúde (Figura 1).

Os resultados dos municípios médios assemelham-se aos da média nacional: redução imediata nas despesas totais e nos recursos próprios/estaduais, sem mudanças nas tendências pós-PAF em todas as variáveis estudadas. A heterogeneidade desse grupo de municípios no que diz respeito à estrutura do sistema de saúde e às características demográficas e econômicas^14^ dificulta a interpretação desses resultados. Sem perder isso de vista, destaca-se que eles tiveram as despesas *per capita* mais baixas em todo o período. Ou seja, embora não tenham sido os mais impactados pela PAF, esses são os municípios que mais carecem de aporte de recursos. A ausência do impacto sugere apenas que o problema antecede a PAF.

No que tange aos municípios de grande porte, demonstrou-se que despesas totais em saúde após a PAF só alcançaram os níveis de 2015, cerca de US$ 185 dólares *per capita* ao ano, em 2019. É razoável propor, dessa maneira, que o maior impacto da PAF se deu sobre esse grupo, uma vez que os municípios pequenos e médios recuperaram os níveis de suas despesas um ano antes.

A queda na tendência de recursos transferidos pela União tem a maior participação nos prejuízos dos municípios grandes. O aumento do volume de recursos alocados por emendas parlamentares (EP) destinados à saúde a partir de 2016 explica em parte essa mudança. Conforme constatam Piola e Vieira^29^, enquanto 1,7% do total de gastos federais com saúde em 2015 era oriundo de EP, esse percentual superou os 7% a partir de 2016. Cerca de 75% desses recursos transferidos para estados e municípios, principalmente os pequenos, devido à preferência dos parlamentares em alocar recursos em locais de maior proximidade com a população beneficiária^29,30^.

Embora essa destinação seja, em parte, desejada, uma vez que esses municípios são mais vulneráveis (Tabela 1), a distribuição desses recursos não se dá de forma equânime entre os municípios, o que pode gerar desigualdade^30^. Além disso, o aumento da parcela de gastos alocados por EP, junto ao congelamento de gastos com saúde no orçamento federal, significa uma redução da parcela de alocação própria pelo Ministério da Saúde^29^, o que implica uma diminuição de seu poder indutor^31^. Ademais, estudos têm destacado uma falta de transparência orçamentária dos recursos destinados por EP^29,30^.

À exceção dos municípios de grande porte, os recursos transferidos pela União foram menos impactados pela implementação da PAF. Estudo anterior^12^ demonstrou que o percentual de recursos federais destinados à saúde transferidos diretamente aos municípios permaneceu relativamente estável desde 2004, embora os valores *per capita* tenham aumentado. Nesse sentido, ressalta-se a importância de regras que estabelecem um financiamento regular dos serviços de saúde, como o Piso da Atenção Básica (PAB). O volume considerável de receitas provenientes dessas normas^12^ contribuiu para amenizar o impacto da PAF no financiamento da saúde.

Investigar quais setores dos serviços municipais foram mais impactados, considerando as particularidades demográficas de cada município, mostra-se necessário em estudos futuros. Sabe-se que a implementação da PAF influenciou na perda de qualidade dos serviços relacionados à saúde bucal^3^. Além disso, também é mencionada na literatura uma diminuição no acesso a medicamentos fornecidos pelo SUS devido à redução dos gastos nas esferas municipais e estaduais 2015 e 2016^32^.

Uma questão indiretamente levantada pelos resultados diz respeito à dependência fiscal dos municípios em relação aos gastos em saúde. Os municípios de pequeno e médio porte experimentaram uma diminuição de despesas com recursos próprios/estaduais, relativamente aos recursos oriundos da União, indicando um crescimento da dependência de recursos federais para gastos com saúde. Cruz et al.^5^, ao analisarem as receitas municipais, observaram um crescimento expressivo das receitas não próprias em relação às próprias a partir de 2016, especialmente entre os municípios de pequeno porte, o que sugere que o crescimento da dependência fiscal não se limita à área da saúde.

A disponibilidade de recursos para os municípios é essencial para a descentralização do SUS e fortalecimento da APS. Nas últimas décadas, o aumento de recursos públicos destinados à saúde ocorreu pelo crescimento de gastos municipais, sustentado, principalmente, pela destinação de recursos próprios^12^. No entanto, os resultados deste estudo evidenciam que esses recursos são suscetíveis aos ciclos econômicos e às decisões políticas do governo federal. Desse modo, corrobora-se a ideia de que os municípios carecem de uma fonte de arrecadação tributária mais regular e estável^27^.

Quanto às capacidades de arrecadação dos municípios brasileiros, cabe destacar ainda que, enquanto este artigo está sendo escrito, se encontra em tramitação o Projeto de Emenda à Constituição n^o^ 45, de 2019, que propõe mudanças nos mecanismos de tributação em todas as esferas do Estado brasileiro.

Os resultados do presente artigo devem ser interpretados com cautela. Os modelos de ITS mostraram que a implementação da PAF não teve impacto estatisticamente significativo nas despesas totais de saúde a médio prazo nos grupos municipais analisados, apesar de um leve declínio ter ocorrido em todos eles. Na melhor das hipóteses, pode-se inferir que tais despesas estagnaram a partir da PAF.

Deve-se considerar que o Brasil tem gastos públicos com saúde baixos em comparação com países de renda semelhante e que não possuem sistemas universais^13^. Argentina e Chile, por exemplo, em 2015 tiveram despesas públicas maiores em percentuais do produto interno bruto (cerca de 1%) e *per capita* (40% e 50% maiores, respectivamente)^13^. Contrastando a realidade com os ambiciosos princípios do SUS, torna-se evidente que agendas de cortes, ou mesmo de estagnação, de gastos públicos, são incompatíveis com a efetiva realização do nosso sistema de saúde^13^.

Uma importante limitação deste estudo é derivada da decisão de não utilizar o indicador de “Participação das transferências para a Saúde em relação à despesa total do Município com saúde”, disponibilizado pelo SIOPS. Essa escolha foi feita devido às alterações nas codificações de receitas e despesas do indicador nos anos de 2016, 2017 e 2019, conforme esclarecido pela Coordenação do Sistema de Informações sobre Orçamentos Públicos em Saúde (CSIOPS/CGES/DESID/SE/MS), em resposta à indagação dos autores. Essas mudanças dificultam uma análise longitudinal precisa da participação dos estados e dos gastos próprios em saúde nos municípios.

Outras importantes limitações devem ser destacadas. A primeira decorre da qualidade dos dados disponibilizados pelo SIOPS, que tem sofrido críticas acerca de sua confiabilidade e completude^33^. Neste estudo, a incompletude dos dados foi uma característica que se fez presente, especialmente nos municípios pequenos, o que pode ter superestimado as suas despesas. Uma última limitação diz respeito ao tamanho da série temporal analisada. O pequeno número de pontos no tempo dificulta a identificação de tendências significativas ou padrões sazonais, a estimação adequada dos parâmetros de autorregressão (AR) e média móvel (MA), além de aumentar a sensibilidade dos modelos aos *outliers*^19^.

Por outro lado, o estudo destaca-se por estimar em abrangência nacional o impacto da PAF no financiamento da saúde dos municípios a partir de ITS, um método quase-experimental indicado para estudos de avaliação de impacto de políticas públicas^19^.

Embora a PAF tenha sido interrompida em decorrência da pandemia, ela ainda ocupa espaço relevante na agenda pública. O Brasil de 2015 a 2019 configurou-se como um laboratório social, proporcionando uma oportunidade para avaliação e denúncia das consequências da austeridade no SUS e na saúde da população. O papel da política fiscal para a estabilização da economia não pode servir de argumento para a supressão de gastos de saúde, haja vista que esses possuem efeitos redistributivos e multiplicadores^34^. Conforme sinaliza Ocké-Reis^34^, a criação de um novo marco fiscal deve considerar a necessidade de aumento dos gastos públicos com saúde para pelo menos 6% do produto interno bruto, com vistas a uma aproximação dos pressupostos do SUS com a realidade concreta.
